# Charity

**Published:** 1903-10

**Authors:** 


### EDITORIAL NOTES AND COMMENTS.

The Business office of THE JOURNAL-RECORD is No. 1007-1008 Century Building.

The Editorial office is 318-319-320 The Prudential.

Address all Business communications to Dr. M. B. Hutchins, Mgr.

Make remittances payable to THE ATLANTA JOURNAL-RECORD OF MEDICINE.

On matters pertaining to the Editorial and Original Communications address Dr. Bernard Wolff, Atlanta.

Reprints of original articles will be furnished at cost price. Requests for the same should always be made *on the manuscript.*

We will present, post-paid, on request, to each contributor of an original article, twenty (20) marked copies of THE JOURNAL-RECORD containing such article.

### CHARITY.

It is as useless to fight against the interpretations of the ignorant as to whip the fog.—*Geo. Elliot.*

It is not a very laudable characteristic of humanity that the value of free services is held commensurate with the price. This phase of human nature, based on ingratitude, is conspicuously directed against the medical profession. Do the many who so lightly in conversation bring to trial the city hospital and other medical institutions of an eleemosynary nature realize what the city would be without them, or that every doctor connected with them gives his laborious services absolutely gratuitously to the indigent sick that crowd the wards? Have we a free legal board of relief where pauper litigants can find representatives who will lend them skilled aid in their difficulties? Because a man has no money to buy food and raiment do the merchants supply his needs? Aside from the shrill vaporings of the callow rhetoricians who obstruct the streets on Sunday afternoons is there really any free preaching, that the soul thirsty for the truth may drink without the subject of pew rent and church sustentation being broached?

No, the doctors bear most of the poor man's burden, and sometimes the weight of the burden galls the withers.

But these are merely a few of the immemorial infamies that are brought against the doctors. They are hoary injustices that are even time-honored, and if any doctor should have the temerity to exercise his right as a tax-payer and a bread-winner to turn a deaf ear to penniless appeal the scorn of the just would hound him out of the community. The relief of the worthy distressed is a high privilege and a pleasing utilization of professional accomplishment; but the bestowal of it must be left to the discretion of the physician himself. The public strains the quality of mercy and has come to regard as its just due what is no more than a concession from motives of humanity on the part of the physician to those who are unable to pay for his services. It is time for the medical profession to get together and keep together and to formulate irrefrangible rules of conduct that will protect it from injustice, injury and obloquy.

Organization for defensive purposes is the order of the day. Apart from mawkish sentiment about the noblest of all professions, medicine is as much a business as any other avocation, intellectual or manual, and as much in need of protective alliance as are they.

